# A scale-free universal relational information matrix (N-space) reconciles the information problem: N-space as the fabric of reality

**DOI:** 10.1080/19420889.2023.2193006

**Published:** 2023-05-11

**Authors:** William B. Miller

**Affiliations:** Banner Health Systems, Paradise Valley, AZ, USA

**Keywords:** Cognition, consciousness, Markov blanket, N-space, N-space episenome, pervasive information field, self-reference, sentience

## Abstract

Cellular measurement is a crucial faculty in living systems, and exaptations are acknowledged as a significant source of evolutionary innovation. However, the possibility that the origin of biological order is predicated on an exaptation of the measurement of information from the abiotic realm has not been previously explored. To support this hypothesis, the existence of a universal holographic relational information space-time matrix is proposed as a scale-free unification of abiotic and biotic information systems. In this framework, information is a universal property representing the interactions between matter and energy that can be subject to observation. Since observers are also universally distributed, information can be deemed the fundamental fabric of the universe. The novel concept of compartmentalizing this universal N-space information matrix into separate N-space partitions as nodes of informational density defined by Markov blankets and boundaries is introduced, permitting their applicability to both abiotic and biotic systems. Based on these N-space partitions, abiotic systems can derive meaningful information from the conditional settlement of quantum entanglement asymmetries and coherences between separately bounded quantum informational reference frames sufficient to be construed as a form of measurement. These conditional relationships are the precursor of the reiterating nested architecture of the N-space-derived information fields that characterize life and account for biological order. Accordingly, biotic measurement and biological N-space partitioning are exaptations of preexisting information processes within abiotic systems. Abiotic and biotic states thereby reconcile as differing forms of measurement of fundamental universal information. The essential difference between abiotic and biotic states lies within the attributes of the specific observer/detectors, thereby clarifying several contentious aspects of self-referential consciousness.

Reality is merely an illusion, although a very persistent one. Albert Einstein

## 1. Introduction

A common assumption is that physics contains much that can be leveraged to understand biological organization, structure, and interrelationships. Nonetheless, there should be circumstances in which biology might inform physics by offering alternative avenues for the latter’s productive interrogation. Accordingly, a hypothesis is presented asserting a universal scale-free relational information space-time matrix (N-space). The N-space concept has been applied to biotic systems as a source of biological order [1–3]. It is now further proposed that N-space can be equally applicable to abiotic states, thereby licensing a unifying frame from which biological N-space would derive. In this framework, the measurement of information upon which biological order depends is an exaptation of a differing mode of information measurement in abiotic systems. To maintain that assertion, an exploration of the particulars of information and how that relates to abiotic and biotic states and universal properties is crucial.

Exactly how information interrelates to matter and energy remains highly debated, although some have insisted that they are all interconvertible [4]. Though many physicists believe that matter and energy are primary and information is merely derivative, others assert that information has its own ontological character since only information makes a “causal difference to our world” ([5] p. 8). Furthermore, that causal information is relevant even in the quantum realm as a quantum wave function represents all that is known about a quantum system. When an observation collapses the wave function, the trajectory of the subsequent evolution of the quantum system is affected as a difference [5].

Thus, for many, a cohesive concept of information is conceived as a ”difference”, implying an observer [6]. This interconnection between a difference and an observer is crucial since observer-based information is related to measurements. Necessarily, as a basic physical principle, any measurement entails an interaction between an observer and an observed object [7]. Accordingly, if matter, energy, and information are interconvertible, information can be productively conceived as a universal elementary observer-dependent substratum. Thus, in a universal framework, information can be conceived as the fundamental aspect of reality as the corresponding observable interrelationship between matter and energy and operating across both abiotic and biotic states. Furthermore, in such circumstances, information constitutes the only knowable reality as the explicit observable interrelationship between matter and energy. Consequently, for all observers, information is the fundamental component of any universal fabric. If so, the corresponding question is whether or not there are non-living observers.

The universe has order based on various fields as products of the interactions of matter and energy. These fields, such as gravity, act locally but are now understood to have nonlocal correlations related to entangled coherent quantum variables [8,9]. Furthermore, each of these fields is information in context. Pertinently, the particles in these fields do not carry the information of the quantum state. Instead, that content resides within the collective wave function of a system of connecting coordinates as coherences in space-time [10]. Consequently, particles, energy, and fields constitute an indivisible whole that can be considered an interconnecting universal informational matrix whose coherences, and differences, contribute structural order.

N-space is conceived as the totality of universal matter–energy interactions whose differences are capable of detection by abiotic or biotic entities. Therefore, N-space is the universal connections between matter and energy, which, as will be explained, should be considered information. It is argued what matters in construing any reality is what can be observed or measured. Matter and energy are real, but they can only be known through interactions between them. Any interrogation of any universal reality must energize this type of interaction. Consequently, N-space is consonant with a universal structure, representing the substrate of connections between matter and energy that can become information depending on context. Consequently, the universe can be construed as a form of information field [11].

In trying to comprehend the universe and our place within it, the issues of information flow have received less scrutiny than energy flow, even across biological disciplines [12–14]. In emphasizing that point, Marijuán and Navarro [15] offer a 2008 quote by Paul Nurse “Perhaps the most pressing need is to develop the appropriate theoretical approaches to analyze the management of the information flow and to investigate the logic systems that are responsible for that flow.”

Given the preceding, establishing a cohesive platform for information flow across biotic and abiotic systems centers on the interlinked issues of what constitutes information and what qualifies as an observer capable of measurement, thereby conferring meaning. To resolve this, many scientists and philosophers have insisted that only a conscious observer can satisfy these conjoining requirements, often with the further assumption that only humans embody true self-referential consciousness [16–19].

An alternative resolution to this contentious issue is based on a conception of universal information as the fundamental universal reality, which naturally implies the presence of universal observers. To sustain that assertion, a novel approach to information connecting abiotic and biotic states is elaborated. Furthermore, three linked propositions are defended:

- There is information in abiotic states.
- Abiotic systems are capable of conditional measurement.
- Information in abiotic systems has meaning.

From these particulars, an unorthodox interpretation of the emergence of biological information measurement as an exaptation from abiotic systems through N-space partitioning is detailed, thereby better explaining the origin of biological order. Finally, and deriving from the above, the general conditions for self-referential consciousness embodied in the cell as an exclusive partition of universal information are explored, emphasizing that all living information is self-produced.

## 2. Current concepts of information

The only point about the concept of information that is likely to achieve near unanimity is that a formal, precise definition of information is elusive at best. Lombardi et al. [20] introduce their pluralistic views on polysemantic information by aptly quoting St. Augustine’s characterization of time in book 11 of his *Confessions*: “What, then, is time? If no one asks me, I know what it is. But if I wish to explain it to one that asketh, I know not.” ([20] p. 1248). Moreover, many believe that information has no real meaning as a stand-alone concept [21]. Küppers [22] maintained that information only exists when there is a preexisting sender–receiver relationship that begins in the living state at the level of biological macromolecules from which all life issues. Consequently, the concept of information pertaining to physical interactions, such as interactions between particles, would be totally alien to any concept of information in the living state [21].

It is instructive to note that one of the hurdles in applying the information concept to biology is that it so closely mirrors the muddle that clouds other biological concepts such as speciation and consciousness. In each of those latter instances, terms are deployed with vagarious and even idiosyncratic flexibility. Consequently, there is no cross-discipline dominant consensus about what those terms precisely mean, and it is just the same regarding information. Part of the problem is that each of those varied definitions is only rigorously applicable within specific disciplines and, accordingly, has corresponding limitations. For example, the well-known Shannon information concept is not a specific theory of information. Instead, it is a theory of communication employing no explicit definition of information [23]. Schroeder [23] points out that the various meanings of information can be in conflict depending on whether that term is being used to denote information as representation, conduit metaphor, used in a linguistic sense, or data computation. There are significant disparities even when confined within the narrower parameters of mathematical or statistical information.

Beyond Shannon’s widely accepted formalism, Fisher [24], suggested a definition conceived as random variables influenced by an unknown parameter that affected measurable outcomes. On the other hand, algorithmic information measures the length of the shortest computational program that produces a string on a hypothetical Turing machine. In contrast, quantum information is a statistical measure of von Neumann entropy judging the quantum resources needed to accurately encode the state of the originating source [20].

The colloquial term ”information” is typically used to refer to non-mental, user-independent contents such as databases or encyclopedias that are objective and mind-independent [25]. Floridi ([25] p. 127) suggests that “information is data that has been processed into a form that is meaningful to the recipient, or information that equals data plus meaning, or information is data that has been received, interpreted and assimilated by the recipient of the message.” In these terms, data are a ”bit” as a unit of information, described as a “single difference” [26]. MacKay ([27] p. 161) conceptualized the functional meaning of that conception of information as “a distinction that makes a difference”, which Bateson [28] more notably changed to the often quoted, “ … ….the elementary unit of information – is a difference that makes a difference” [25,26,28]. Conspicuously, in biosemiotic applications, symbols are regarded as the functional units of information rather than bits [26].

Schroeder ([23] p. 2) cites MacKay’s well-known representational view of information: “Every piece of information has the characteristic that it makes a positive assertion and at the same time makes a denial of the opposite of that assertion.” Pertinently, MacKay [27] (p. 161) left the concept of representation that was central to his thoughts purposefully broad since “By representation is meant any structure (pattern, picture, or model) whether abstract or concrete, of which the features purport to symbolize or correspond in some sense with those of some other structure.” Again, Schroeder [23] emphasizes that this definition is meant to confer structure to information. However, MacKay stressed that structural information is not explicitly related to the number of elements within it or their pattern but is inclusive of “the possibility of distinguishing between them.” Furthermore, and quite pertinently, Bateson had included a time element in his original definition of information as a “difference that makes a difference,” in an original articulation as “information is any difference that makes a difference in some later event” [23].

Others have followed MacKay’s approach that there can be no information without representation or that information is something that adds to a representation [23]. Dretske [29] offered another interpretation, indicating that “information is a commodity that, given the right recipient, is capable of yielding knowledge.” Fresco et al. [30] further insisted that functional information is a “triadic relation amongst a receiver, a difference-maker (e.g., a communicatory signal), and an object/feature/state of affairs.”

Based on the preceding, most scholars assume that there is no actual information within abiotic states. Inanimate objects interact, but for many scholars, “ … ….information appears in the Universe only wherever and whenever life appears. In the abiotic world, there is no information, unless there is an interaction with a living organism” ([21] p. 36). Notably, physicists disagree. In quantum mechanics, information is physical as it is encoded in the state of a quantum system [31].

Given the variety of opinions, it is necessary to settle on one basic definition of information in order to further elaborate and defend the hypotheses being presented. Accordingly, information within this construct adheres to the generally accepted formulation of MacKay and Bateson as a ”difference that makes a difference”. As will be explained, this implies the further entailment that such a definition of information is observer-dependent.

## 3. A holographic relational information universe

The concept of a universal information field as the basis of consciousness has been previously argued based on an underlying nonlocal quantum field exhibiting holographic properties (see Meijer [32,33], for a comprehensive list of relevant papers). Conceptually then, the universe can be conceived as a hologram based on the Holographic Principle, contending that the three dimensions of space we perceive are our representation of two-dimensional space. Recent experimental evidence has supported this perspective [34]. Furthermore, Fields et al. ([35] p. 72) specifically argue that the holographic principle is “not just a fundamental principle of physics, but of all science”.

Glattfelder [36] has proposed that information is a fundamental universal reality and has demonstrable holographic properties within that type of universal structure. In this specific context, information is considered a form of computation, and Glattfelder [36] argues that computational information is a measurement in holographic space-time where information is an emergent property of quantum entanglement.

Many have stressed that information is the fundamental constituent of the universe, constituting its actual fabric, whether involving inanimate particles or living structures [37]. Lloyd [38] modeled a quantum computing information universe as an information processing architecture. Whitworth [39] further insisted that the physical world is a virtual one created by information processing. Accordingly, the concept that information is a basic property of the universe has a long provenance.

Stonier [40] linked energy to information, noting its capacity to do work and organize any impacted system. Consequently, energy and information might be deemed fully interconvertible. However, physicists have generally ignored that relationship as not necessarily entwining. Accordingly, for many phenomena, concepts of potential energy and entropy have been used exclusively to reconcile the law of conservation of energy [40]. In contradistinction, Stonier [40], argues that the conservation law should entail both energy and information to properly balance the equations, proposing that there is a measurable formulaic relationship between the two.

Norbert Wiener thought differently about the relationship between information, matter, and energy, stating, “Information is information, not matter or energy. No materialism, which does not admit this, can survive at the present day” ([41] 1961, p. 132, from [42]). Nonetheless, Wiener concluded that information has universal primacy, regarded information as the “most pervasive and unique element that is abundant in the universe” as a preexisting, fundamental universal quantity. In consequence, this stance has found a conducive home in many diverse fields, such as biosemiotics [42].

When the universe is placed within an informational context, it is typically framed as universal cosmic consciousness or, at least, an epistemic agency [17,43,52]. In such a universal informational fabric, even atoms can accumulate information [44]. Dodig-Crnkovic [43] summarized Alan Turing’s vision of an info-computational universe, emphasizing an essential interplay between informational structure and computational processes that constitute a type of informational self-structuring. In this framework, “the process of computation implements physical laws which act on informational structures” [43]. In a reciprocating manner, the process of computation affects physical forms in a “form-changing/form-generating process”. Within this conceptualization, physicist Stephen Wolfram found a fundamental equivalence between matter and information: “[M]atter is merely our way of representing to ourselves things that are in fact some pattern of information, but we can also say that matter is the primary thing and that information is our representation of that. It makes little difference, I don’t think there’s a big distinction – if one is right that there’s an ultimate model for the representation of universe in terms of computation” ([43] quoting Wolfram).

In his pivotal book, *Wholeness and Implicate Order*, David Bohm presented a model of holographic universal order that criticized the limitations of the Cartesian dualism and insisted that quantum physics revealed a “universe of unbroken wholeness” [45]. The universe, it was proposed, is indivisibly interconnected through an implicate order whereby every separate space-time location also contains the entire universal structure “enfolded” within it, constituting fundamental reality. Our perception of that reality is the “unfolding” of this implicate order as explicates triggered by subjective human perceptions as a projection of that hidden total implicate order [32].

Central to this perspective was Bohm’s insistence that the universe had holographic features, termed the “holomovement” from which all forms derive [45]. In this holographic universe, every particle and every aspect of each separate universal space-time location is connected to all other parts of the universal matrix in a seamless whole. de Gosson and Hiley [46] argued that the holomovement was the universal fundamental, and all physical states emerge as derivatives of interactions within the holofield as the only true reality. Correspondingly, in *Quantum Reality Unveiled Through Process and the Implicate Order*, Hiley [47] emphasized Bohm’s vision of the holomovement as an active flux with all matter and its interrelationships viewed as explicit features of this fundamental fluidity [45].

Conceptually then, everything emerges from the unfolding of the holomovement, encompassing all potentials as the agency from which all forms and objects emerge [45]. Consequently, our intuitive order of things is inverted. All agency arises from “the conditions of the nonlocal whole and not by the local parts” and it is the universal connections that are paramount [45]. Hiley [47] recounts Bohm’s 1980 mixing experiment, which was meant to illustrate this implicate order through purely physical means metaphorically. That deceptively simple experiment involves two transparent cylinders that can rotate with respect to one another. Glycerin is placed between them, and a spot of dye is placed in the glycerin. When the cylinders are rotated, the dye spot smears and eventually disappears completely. However, if the rotation is reversed, the spot of dye reappears, thus revealing a distinctive hidden order. Despite its appeal to Bohm, the experiment seemed unsatifying to many others.

In Bohm’s radical view, contemporary physics obscures a larger imperative. Meaning is itself implicate throughout the holofield. Meaning enfolds both matter and energy, and accordingly, meaning is more fundamental than matter and energy [45]. Furthermore, both Bohm and the philosopher and mathematician Alfred North Whitehead envisioned a universe with embedded experientiality that also encompassed the abiotic state. From their perspective, all reality is experiential as enfolded meaning [48]. In their terms, the universe is defined through a relational universal “sense-awareness” [49]. Others have maintained similarly, insisting that the universe must be placed in a relational informational frame as a summation of the entire stream of interreactions between matter and energy from which meaning derives [52](Meijer, 2015). Furthermore, that meaning cannot be subtracted from fundamental information, and therefore, there is an embedded dynamic flow of information at all levels

Bohm was dissatisfied with the breadth of his initial field equations, offering a new set of quantum potentials. He detailed a quantum model of the universe in which quantum potentials as wave functions carried active information into a supraimplicate order. This further order represents an overarching field state as a “function of wave functions” that settles the implicate order into non-linear complex structures [18]. In this construct, universal order and consciousness arise from within this supraimplicate realm.

In the contextual framework of a universal supraimplicate information matrix, meaningful information requires the presence of a conscious observer. By implication, only humans were considered genuine conscious agents, and for many scientist-metaphysicians, human consciousness is the veritable, fundamental building block of nature by which human consciousness constitutes universal reality [17,50–53]. In this context, universal holographic space-time is considered to exist on the surface of an entire holographic universal *now* from the inception of an experiential universe [54]. Moreover, at the physical level, the concept of a holographic workspace for human brain-centered consciousness has been previously proposed in a framework of integrated wave energies whose reverberations yield conscious states [52].

Consequently, a substantive academic framework supports the premise of a universal informational fabric whose meaning requires a conscious observer. In apposition, it is alternatively contended that within a universal informational fabric, the necessity of a conscious observer is not a requisite precondition since the act of observation-measurement exists without consciousness as embodied awareness.

Stonier [55] proposed still another perspective: “*Information exists*. It does not need to be *perceived* to exist. It does not need to be *understood* to exist. It requires no intelligence to interpret it [to exist]. It does not have to have *meaning* to exist. It exists.” Three informational theorems were offered supporting this thesis and emphasizing the organizing capacity of information [40]:

1. Every organized structure contains information and no organized structure can exist that does not contain information.
2. Adding information to an organized system makes the system more organized or reorganizes it if perturbed.
3. All organized systems are able to “release or convey information”.

Within this perspective, information is just as much a fundamental property of the universe as matter and energy. In this frame, energy is the capacity to perform work, and information can be usefully defined as the capacity to organize a system. Both can be deemed interconvertible. Yet, both matter and energy are themselves interconvertible. If so, they are all inherently entangled.

The stance being taken here differs from Stonier and others. Information exists and is universal because observation is also a universal attribute, and as will be further explained, the observer need not be conscious. The reason that information can be productively considered the capacity to organize a system is due to that observation-measurement context, which effects that ordering

In Bohm’s view, wholeness was universal, unified, and interconnected through an indivisible implicate order. Whatever physical objects we might observe within the continuum of space-time are representations of the explicate order that has unfolded from the implicate order. The structure of a hologram is instructive in conceptualizing this non-intuitive concept. In a hologram, every local region of a projected three-dimensional image contains that entire image mapped at lower resolution within all other partitions within the entire holographic field. In a hologram or the holomovement, for that matter, “each fragment of the image is not only a part of the whole but also an instance of the whole” [45].

Mackay [56] provides a constructive conceptual frame for universal information content. Information can be well represented by a vector whose space-time dimensionality reflects its structural information content (quality), whereas its vector length represents its quantitative aspect (density). Mackay further developed this framework by supposing “conceptual units of space-time”, wherein greater numbers meant a finer detailing of the relevant information content. Both of these conclusions provide insight into the nature of a universal holographic information matrix. Mackey insists that information acquires meaning only in terms of what it does and how it is situated in information space. This melds with the corresponding concept of pragmatic information as something that affects something, such as permitting the evaluation of the state of a quantum system. This framework also includes information deposited within the active environment or extracted from it and correspondingly applies to both abiotic and biotic states [57].

Roederer [57] argued that pragmatic information distinguishes biological information from Shannon statistical information. The latter represents the minimal number of binary steps to define an entity or process that has no distinct biological context. Instead, living systems use pragmatic information in which information quantity is subordinate to what information actually does, irrespective of the number of bits involved. To a significant degree, according to Roederer, it is this subjective, qualitative aspect of information from which meaning arises.

Accordingly, there are a great variety of opinions about what constitutes both universal information and how the universe interconnects. For our purposes, the universal interactions between matter and energy represent information if observed. Since every interaction between matter and energy yields a difference, that difference can be information to a relevant observer. Accordingly, N-space can be conceived as a universal information space with constituent universal observers. Furthermore, following MacKay, information in universal N-space can be considered an ordinary vector space in which vector position and length reflect both qualitative and quantitative information content with vector intersections corresponding to information density in space-time, conforming to Mackay’s theoretical “units of space-time”, forming a universal information fabric.

Accepting the position of MacKay and Bateson, information should have meaning as a “difference that makes a difference”. This leads to the salient question of what qualifies as an observer? Specifically, can abiotic entities be considered observers so that information be construed as meaningful and therefore capable of participating in an observer-based measurement of those matter–energy differences? It is being proposed that these qualifications can be met when a holographic information matrix can be partitioned as will be explained. Within that construct, observers are both universal and capable of an abiotic form of measurement conferring meaning.

## 4. Conceptualizing N-space partitions

The concept of N-space partitions and their relationship to *k*-space architecture has been previously described in depth [1]. The point of discussing *k*-space is that its architecture provides a framework for understanding the partitioning of universal N-space information, offering a means of conceptualizing how N-space compartmentalization applies to biological systems. Pertinently, this practical imaging technology uses an information matrix that has all of the essential characteristics of theoretical N-space partitions.

In brief review, *k-*space is an information field generated for magnetic resonance imaging (MRI) in living systems and utilized in photoelectron spectroscopy for the investigation of nanostructures in semiconductor electronics [58]. In MRI, *k*-space is a topologically coherent compact, digital space in which there are complete families of other compact sub-compartmental spaces generated as an extension of Fourier space, permitting the mathematical encoding of 2D and 3D spatial information [59]. In this application, *k*-space is a temporary digital space created by electronic data acquisition from which an image of a specific field of view can be formed based on the analysis of time-varying signals. In the case of MRI, these latter are radiowaves of differing frequencies and phases. A data field can be acquired from timing different sequences of frequency-encoded radiowaves that stimulate reciprocal characteristic echoes from the living subject. These can be interpolated among different applied phase-encoding electrical gradients (a gradient in an orthogonal axis) across a main magnetic field to enable spatial location and the acquisition of either anatomic or physiological information.

**Figure 1.**
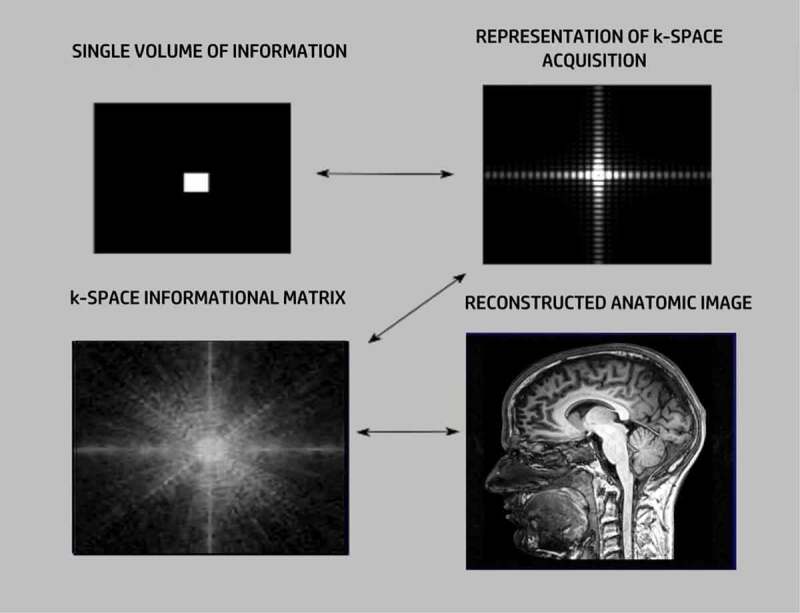
Image formation in *k-space*.

The data acquisition in MRI depends on the presence of a powerful external magnetic field, representing a Z-axis coordinate, capable of inducing coherent precession of protons (mainly hydrogen atoms bound in water) within the investigative field as dipole–dipole interactions. If the radio waves are at the same frequency as the proton’s precessional frequency (resonance), the proton’s magnetic moment flips away from the Z-axis. That precessional resonance frequency, termed its Larmor frequency, is proportional to the main magnetic field strength. When the RF pulse is interrupted, the precessing protons in resonance slowly return to their original state, yielding measurable time-decay frequency encoding curves that can become localizing information. Precession can be conceptually considered akin to the wobble of a spinning top. If that spin is deflected, it will return to its primary axis of rotation. The consequent release of energy can be detected by an antenna and mathematized through a Fourier transform to become a spatial image by imposing an orthogonal electric field gradient placed across the main magnetic field from which positional information can be interpolated. The acquired frequency-encoding data is stored in *k*-space, which is a mathematical spatial map of that accumulated data, which can be assigned a grayscale from which an anatomical image can be reconstructed (Figure 1).

In an MRI acquisition, all points within the field of view have a corresponding representation in holographic *k-*space informational architecture. Each volume of information results in a unique *k-space* partition representing a combination of frequency and phase encoding that also fills the entire *k*-space informational matrix. In *k-*space, information obtained along orthogonal axes permits spatial dimensionality, which can be transformed into a biological image. This same holographic process has been applied to a new understanding of morphogenesis and cellular developmental patterning.

Crucially, no one-to-one correspondence exists between any point in *k*-space and the resulting total image. Instead, in *k*-space, every point has contributions from every other point and every *k*-space sub-compartment receives a contribution from every point in *k*-space [60]. Consequently, every *k-*space point has summary elements from other points across the entirety of *k*-space. Accordingly, *k-*space has constituent holographic features upon which universal N-space can be modeled.

Furthermore, and significantly, in a *k*-space matrix, the compartments in the center of the imaging field have the highest information density since their informational contributions are greatest across the field. Peripheral compartments still have some data from every *k*-space compartment, but the information density at the periphery is less. This same phenomenon is pertinent to quantum relationships. Any two points in space-time are entangled no matter how far apart they are. The difference is that the closer they are, the more they are entangled and the more likely they are to be available to settle superpositions.

Pertinent to conceptualizing N-space partitions, the nature of the data acquisition for each individual *k*-space partition within the larger *k*-space matrix permits a reverse reconstruction. It is possible to take the MRI image and reverse reconstruct a *k*-space matrix almost identical to the one used to create the image. Consequently, *k*-space functions as a highly flexible read–write architecture.

A notable aspect of *k*-space particularly applicable to N-space is *k*-space’s symmetrical effects as inherent informational redundancies [1,60]. MRI scanning is time intensive, and one way to economize without any substantial loss of resolution is through leveraging the symmetrical properties of *k*-space. Any *k*-space data point contains information from all other *k*-space compartments. In MRI *k*-space, positive spatial frequencies have symmetrical negative frequencies that also populate the *k*-space matrix. Useful information can be derived from those symmetrical values. Relevantly, this phenomenon resembles the concept of antipodal information and information on the adjacents, which are among the reasons for informational ambiguity in the living state [12,61].

Antipodal information is those aspects of information that extend beyond what can be directly observed or sensed [62]. A simple example is a sphere like a soccer ball that might convey different information content to separate observers on opposite sides. Each of the observers is presented with en-face information. However, there is antipodal symmetric hidden information on the opposite side, which in *k*-space or N-space would be similarly encoded but opposite in value.

The information on the adjacents has been modeled by Marijuán et al. [12], conceptualized as a spread of information that can be brought closer together, thereby decreasing informational uncertainties. This concept can be visualized as a dimple on the surface of a golf ball. A straight path taken across the dimple is shorter than a trip around its edges and would be considered a narrowing of adjacents with a concomitant decrease in uncertainty, thereby constituting higher informational validity. From these relationships, it can be intuited that living organisms take similar shortcuts in their decision tree in attempting to narrow informational uncertainties.

Two other field concepts can be used to explain the N-space concept as a universal information field and its relationship to *k*-space architecture. A nilpotent quantum system has been used to explain neuronal organization in the visual cortex since nilpotent quantum states and their environments mirror one another [63]. Nilpotent structures comprise universal rewrite systems within a systematized computational mathematical framework with a “zero totality alphabet”. This alphabet has constitutive features that include self-similar branching, scale independence, and holism at all scales [63]. A zero totality alphabet within a nilpotent state is a commutative mathematical tool that begins from a basal value, permitting successive episodes of mirror-imaging after rewrite. After each rewrite, the reiterative pattern starts again. Thus, there is never a return to the same baseline, but a reticular set of relationships is reiterated. In effect, each rewrite is a new ”zero” within the alphabet serving as a new initiating reference point. However, each still has a direct relationship to all prior iterations in the chain [63]. This can be reasonably analogized to evolutionary reproductive generations through successive episodes of terminal addition in a reticular pattern that retains critical attributes of its prior states [166].

The concepts of Euclidean space help explain theoretical N-space partitions [1]. The Euclidean space is a multidimensional topological space in which each dimension has its own designated affine coordinate, also termed “*n*-space”, as a mathematical space. An affine is not strictly a vector but a combined vectoral displacement representing an ordered pair. Notably, just as with nilpotent structures, there is no requirement for a specific categorical origin of any affine. A series of successive calculations start again when a certain point is chosen, becoming the new origin. In mathematical terms, Euclidean planes convert to a higher number of dimensions, dependent on subtle variations of vector (affine) rotation.

Accordingly, vectoral shifts correlate with different dimensions of informational ambiguity, such as information on the adjacents. The degree of displacement of the rotational vector analogizes with degrees of a “difference that makes a difference”, as greater or lesser degrees of informational validity. Informational density can be considered to correspond to the intersection of vectors. Furthermore, and notably, any improvement in vectoral informational validity directly links to decreases in variational free energy, which can be modeled as enhanced predictive value through inference and a minimization of surprisal [64].

With that as background, N-space represents a universal relational informational fabric whose partitioned features resemble those of both *k*-space architecture and Euclidean vector space. Although *k-*space in MRI is typically populated by positional information, serial observations can be mathematically linked to track particle motion and momentum pertaining to physiological processes as a *k*-trajectory [65]. Since the N-space partition pertains to a self-referential observer with time-dependent observations, any vectoral information in an N-space partition would reflect both position and momentum and have observer-dependent dimensionality. Consequently, N-space partitions would be envisioned as an affine vector space whose dimensional particulars relate to observer status. Since it also conceived as a read–write information architecture, the characteristics of MRI *k*-space in which sub-compartments of information reside within a mathematical matrix also pertains. *K*-space and Euclidean space are not the same but have complementary mathematical characteristics, insofar as any Euclidean space of *n* dimensions can be extended to define a corresponding *n*-dimensional *k*-space [66].

Significant though, N-space and *k*-space are merely being considered in analogous terms. Nonetheless, the concepts presented about *k*-space, Euclidean *n-*space, and nilpotent quantum structures help to explain the general dynamics of N-space architecture as a universal informational fabric that can be partitioned according to the attributes of observers. Universal N-space can be partitioned into sub-compartments, and just as in *k*-space, each N-space partition is still related to the whole, again akin to *k*-space. Consequently, in the living condition, N-space partitions can be conceived as representing the totality of its potential informational inputs similar to a constrained field-of-view that permits the living organization of information. Notably, it is advanced that N-space sub-compartments underpin biological information space-time for all organisms at all scales.

Each of the concepts of N-space that have been presented has direct pertinence to biological mechanisms. For example, the Euclidean affine structure has been deployed for modeling phenotypic development, morphospaces, morphometrics, and evolutionary genetics, validating its use in biological contexts [67]. Furthermore, Rowlands and Rowlands [167] argue that there is a nilpotent quantum universe and indicate that many human brain functions are similar to a nilpotent system. Consequently, the concept of a reiterative fresh “cardinality” based within a historical sequence shared by both Euclidean space and nilpotent mechanics with its “zero-totality alphabet” matches biology as a series of successive articulations with a subsidiary memory of its past within its next re-elaboration [1]. For instance, every unicellular reproductive recapitulation among multicellular eukaryotes is a rewrite of the next macroorganic elaboration that must remain consonant with both basic cellular rules and recent productive adaptations. Significantly then, in the N-space framework, each recapitulation is a re-centering of its constitutive heritable read–write N-space informational architecture as its effective partition within universal N-space as both a new cardinality and a summation of all priors.

As originally envisioned, N-space was the totality of all potential sources of universal information and its partitions were only applicable to living organisms as their constitutive summation of all potential sources of cellular information through its senomic informational architecture [1]. It is now proposed that the N-space model can be extended and productively applied to abiotic systems. In this revised framework, N-space is further conceptualized as encompassing all material and energetic aspects of the universe within a scale-free unifying relational information matrix which accordingly also applies to abiotic entities.

## 5. N-space as a unitary universal relational information framework

The most curious part of the thing was that the trees and the other things round them never changed their places at all: however fast they went, they never seemed to pass anything. “I wonder if all the things move along with us?” thought poor puzzled Alice. And the Queen seemed to guess her thoughts, for she cried, “Faster! Don’t try to talk!”

Lewis Carroll, Through the Looking Glass

Some have contended that information is physical [36,68,69]. Still, there are important distinctions between potential information as superimposed implicates and actual information with explicate physical meaning. The assumption has been that the latter only results from a conscious observer/participant interaction. In an effort to resolve this gulf, an alternative framework for abiotic information is presented, permitting a unifying integration with those aspects of information that are known to be operative within the living state.

Lombardi et al. [70] note that information has been arbitrarily and rather loosely classified into several forms: a) semantic information that connotes “reference, meaning, and representation”, pertaining to a change of state of affairs directed to other things, b) mathematical/computational information concerned with the formal properties of different kinds of systems, concentrating on issues such as defining the compressibility of sequences of states of a system, its storage, or its role in the correlation between the states of two systems, independently of the meaning of those states, and c) epistemic information that provides practical knowledge and modifies the understanding of those who receive it, conforming with our everyday, casual view.

In seeking the scope of information that might be constructively applied to abiotic states, a semantic definition of biological information offered by Jablonka [71] based on the prior work of Bateson is relevant. Information consists of reactions between a source and a receiver that can affect either the potential or actual actions of the recipient. Those reactions should stimulate a complex, organized subsequent chain of events dependent on the organizational status of both the recipient and the source. Furthermore, variations in the source must yield a corresponding variation in recipient response [72]. Put more informally, information is something that causes a next state as a reaction. Küppers [22] had previously argued much the same, insisting that for information to exist, there has to be a purpose, evoking a specific change in the recipient. Thus, it can be defended that the only absolute qualification for the presence of information is a sender–receiver reaction coupling between existences that enacts a discretely related change from one to the other dependent on changes in the source. In sum, there must be a conditional interrelationship between participants that makes a meaningful difference. It is now offered that there are particulars within abiotic states that satisfy this requisite for conditional informational interrelationships and, furthermore, that these contribute to organized states.

The N-space framework of a scale-free universal relational information matrix is an essential element of establishing meaningful information within abiotic states. In holographic N-space, information is a fundamental universal attribute as the obliged interrelationship between matter and energy in which every interaction has some element of representation at all other universal locations. It is advanced that abiotic interactions represent meaningful information by contributing to universal N-space and through a novel concept of a sub-compartmentalizaton of N-space in abiotic states. As will be explained, partitioning of N-space creates conditional attributes for the interactions among abiotic participants sufficient to be deemed meaningful information.

This concept of the compartmental partitioning of N-space information space-time has already been productively applied to biology, helping to explain the origins of multicellularity, morphogenesis, and developmental patterning [1,73]. Within the N-space framework applied to biotic states, each cell has its own Pervasive Information Field (PIF). This information field represents a summation of all possible informational inputs to any cell as its specific interrelational informational network, representing an essential component of cellular information management. Cellular PIFs aggregate into corresponding multicellular N-space episenomes as a summary field of overlapping PIFs. In turn, every N-space episenome links both to the respective N-space episenomes of other macroorganic entities and also to outward holographic N-space. Consequently, PIFs and their corresponding N-space episenomes represent cohesive informational platforms at scale for the concordant measuring assessment of information, permitting seamless multicellular information management. It is now argued that applying this same concept of information compartmentalization to abiotic states is explanatory.

In an N-space abiotic framework, large physical structures, such as galaxies with 100 million constituent stars on average, represent compartmentalized nodal zones of N-space information density within universal N-space. All galactic structures are clusters of particles and energy, and energetic interactions between them are information given an available observer. In this hypothesis, universal N-space can be partitioned into compartmentalized N-space informational nodes wherein each organized physical structure, such as a galactic cluster, is regarded as conditionally independent of others, modeled based on conforming to abiotic Markov blankets and boundaries. These Markov blankets and Markov boundaries have been previously defined as relatively contained units that can occur at nested spatial scales and are linked to others through parents, children, and parents of those children. Thus, a Markov blanket is a set of nested information-encoding states separated from the environment [74]. Furthermore, Markov blankets are conditionally independent of one another and can be partitioned into receptive and active states that mediate differences between what is within the Markov boundary and its external environment [35]. The boundary for a node is variable, and every Markov blanket and boundary are directly impacted by neighboring nodes and their relational network with parental nodes. Markov blankets have been used to account for spontaneous pattern formation and the emergence of complex living structures, including multicellular organisms [75]. A critical attribute of a Markov blanket in living states is their physical boundaries are conditionally dependent on neighboring states, and these relational dependencies between states determine their location in space-time [76]. Significantly though, within Markov blankets, the internal random dynamic system is conditionally independent of the dynamics of its external environment [35].

Within this construct, it is now argued that each abiotic Markov blanket constitutes a separate individual variable non-inertial quantum rotating frame with respect to all others within the universal holographic informational framework. Accordingly, abiotic observers become conditionally independent of others in different non-inertial nodes. Each will experience any quantum interactions with relative independence from abiotic observers in other nodes due to variations among N-space partitions and quantum reference frames. Consequently, information within separate quantum rotating frames derives meaning as it becomes a function of conditional assessments of entanglements and coherences among observer particles in different frames. Accordingly, interactive differences between abiotic particles and energy achieve meaningful informational differences with qualitative as well as quantitative features within their separate partitioned N-space node affecting internal nodal cohesion.

Qualitative differences arise because there are asymmetries in quantum entanglements and correlations between observers in different rotating quantum frames. Yet, within a universal holographic field, all abiotic particles are potential observers. However, their probabilities of participating in an observational settlement of a quantum interaction are conditionally different. Accordingly, not all observers are equally suited to settling any individual superposition dependent on their specific quantum reference frame and its conditional Markov nodal relationship with other nodes. Hence, entanglement differences and coherence distortions directly relate to variations in quantum uncertainties as superpositions between separate reference frames.

Notably, in a holographic information field, there are unlimited widely separated potential participants to settle quantum uncertainties. Each will do so based on the particulars of its interrelationships in separate frames. Any of these may conditionally settle superpositions as a “difference”. Thus, the settlement of entanglements separates from the discrete binary characteristics of the resolution of entanglements within the laboratory frame. Their ”meaning” derives from within these differential settlements based on their situation within a reference frame, obliging differing subsequent quantum trajectories.

Lombardi et al. [20] rejected the notion of meaning in information since the word conveys such a variety of notions that no single concept of information could satisfactorily account. However, that concern can be bridged by defining information as the applicable connective tissue between matter and energy given an observer. Both are constituents of an omnipresent holographic interrelational information matrix that constitutively contributes universal structural order, thus comporting with Bohm’s notion of universal meaning. In that context, any interrelationship that contributes to universal wholeness gains and contributes meaning to the fabric of universal order throughout the entirety.

It is known that quantum entanglement and complementarity are the rule rather than the exception. Untangled, independent states are, in fact, rare since systems are continuously interacting. Furthermore, although the concept of quantum entanglement is generally regarded as precise, there is no universal agreement that this is the circumstance. Raychev [77] notes the difficulties in the quantum measurement of entanglements and their quantum properties since these must be conducted with full diagnostics having probabilities of damage from decoherence and interference.

Consequently, it can be argued that informational meaning exists in abiotic states and that this meaning derives from its conditional, context-related measured assessment of inherent uncertainties in entanglement correlations as its qualitative aspect. Any resolution of implicates as superposition entanglements in information space-time reflects a shift from one previously ordered state into a “next” whose trajectory is not entirely preconditioned.

Although it has been maintained that no observer is necessary to settle quantum entanglements, others disagree [10]. Placing all information into a universal holographic N-space informational matrix with unlimited observers in separate Markov nodes of information constituting separate non-inertial reference frames helps overcome disputes about observer status in abiotic systems. In a vectoral information matrix framework (N-space), separate physical structures constitute distinguishable nodal clusters of information as they are concentrated areas of vectoral intersection. In this context, the meaning of that information is dependent on its observer/detectors, which are dispersed universally within unlimited nodal compartments as there are unlimited physical clusters within the universe. Thus, in universal N-space, there are abundant observers of quantum entanglement asymmetries and coherences, not all of which will participate in the settlement of any quantum phenomena. Significantly, it is directly argued that this discrimination constitutes a measurement since the probability of settlement by any abiotic entity differs among separate quantum relational states.

In a universal holographic matrix, potential observers are everywhere, scattered across billions or trillions of separate quantum reference frames as zones of dispersed informational compaction. Within a universal relational information matrix with its constitutive holographic properties, there are infinite possible observers for any specific entanglement (informational asymmetry). Simply put, there is always an observer somewhere within universal N-space, accounting for the external observable universe.

Accordingly, the universe is information, and that information is the relevant universal reality. The nature of that reality is observer-dependent. Since potential observers are everywhere, but only some participate, the assessment of information has conditional context-dependence. Furthermore, that information has the prospect of being detected by observers in separate non-inertial reference frames. Consequently, their measuring values as probabilities of the settlement of quantum interactions will differ according to the observer context. As these differ, it is advanced that this differential achieves meaning since that participation contributes to the internal status of the dynamic reference frame within that nodal partition of the greater N-space universal informational space-time fabric. The frequency of those settlements naturally correlates with information densities, thereby constituting a form of quantitative meaning contributing to universal order.

It has been previously argued that in quantum systems, there are relevant asymmetries that can be exploited based on the amount of entanglement in quantum systems and the “algorithmic complexity” of prior states [78]. In such a circumstance, it is contended that causal asymmetries within a universal holographic information matrix can be regarded as a proxy for information quality beyond quantity. Furthermore, the informational shifts within the universal informational matrix confer a conditional “arrow of time”. A form of this issue has been previously approached by attempting to reconcile time asymmetry in our universe through the Past Hypothesis. This theory supposes that the universe originated in a state of extremely low entropy and is continuously evolving toward higher entropic states leading to our nominal sense of time [79]. Although thermodynamic entropy and informational entropy are not considered identical, they are indeed closely matched [80].

Physicists are uncovering that basic principles of quantum statistics can be leveraged to clarify the Laws of Thermodynamics [81]. Notably, in an information framework, the Second Law regarding entropic flux need not be conceptualized according to classical probabilities but can instead be approached based on how quantum systems share information, particularly through entanglements. Consequently, increasing entropy is not based just on statistical probability but can instead be viewed as a property of a quantum “resource of information” [82]. Thus, particle-wave duality is information in the context that contributes to a next duality. Pertinently then, it can be entertained that the Second Law of Thermodynamics might have its own antipode that consists of the binding attractional effect of information as a form of universal connective tissue, preventing complete disorganization, helping to maintain universal order and ultimately permitting the living state. This same attractional effect might offer an alternative approach to the still perplexing discovery that the early universe had a far greater number of galaxies than the standard model predicts, and these galaxies are better organized and larger than expected [83].

Wheeler [168]had formulated his well-known “it from bit” concept to support his thesis of an information-participatory universe into a coherent quantum theory uniting information and physics [18]. Within that framework, “…every it – every particle, every field of force, even the space-time continuum itself – derives its function, its very existence, entirely – even if in some contexts, indirectly – from the apparatus-elicited answers to yes – or–no questions, binary choices, bits” (as quoted in [18]). In Wheeler’s construct, that “yes–no” is meant to signify an indivisible bit of information [18]. However, an alternative argument can be advanced that in a relational information context of universal entanglements populated by constituents within myriad separate quantum reference frames due to a partitioning of N-space, the network of entanglements may not be an explicitly yes-no binary system. Instead, it can be conceived that the separation of informational nodes (that relate to galactic structures, for instance) has constitutive uncertainties that convey meaning as ambiguities requiring measurements that emerge from within those collective relationships. Conceptually, particles conditionally “measure” whether their entanglement properties are suited to settle an asymmetry and achieve symmetries and coherences through interrelationships that are not exclusively binary.

In that regard, four phenomena can be deemed applicable. First, in at least some mixed states, experimental evidence indicates that asymmetric speeds in entanglement decay can be considered an entropic inequality in which entanglement might not be considered a pure state [84]. Indeed, quantum state asymmetries are important in quantum information processing, quantum communications, and quantum measurement studies [85]. Furthermore, there are well-acknowledged potential connections between asymmetry and entanglement, at least within special cases [85].

Secondly, matter and energy have their own form of memory. Hysteresis is a common phenomenon occurring when the value of a physical property lags behind changes in the effect causing it. The lag in magnetic induction behind an applied magnetizing force is a familiar example. Furthermore, Mashhoon [86] maintains that although it is commonly assumed in the standard theory that an observer’s acceleration is irrelevant for the purpose of any instantaneous measurement, nonlocal theory implies differently, arguing that this measurement contains a “kernel of the memory of past acceleration” [86].

Third, when information is compartmentalized and placed in a framework of variable field partitions, the informational content of entanglements and the precessional motions of participant particles can shift, affecting observer measurements. For example, in the classic double-slit experiment, the Aharonov-Bohm effect indicates that for electrons subjected to electric or magnetic fields imposed on one side of the axis but not the other, phase relationships are shifted based on electric potentials, changing entanglement probabilities [87]. Naturally, this would have its reciprocal effect on an N-space compartment as an informational shift and an uncertainty relationship. Furthermore, entanglement and precessional asymmetries are affected within reference frames. Internal quantum sub-systems act as imperfect quantum reference frames with imperfect quantum clocks, affecting asymmetries that link to quantum phase transitions [88,89].

Fourth, and of particular importance for this framework, in a quantum rotating frame such as a localized N-space informational compartment quantum rotating system, the state of any localized observable may be in a superposition of two (or even more) states, depending on the observers. Thus, these represent differential entanglement properties between observers in different reference frames, i.e. the same entanglement can yield different observations separating quantum rotating frames [90].

**Figure 2.**
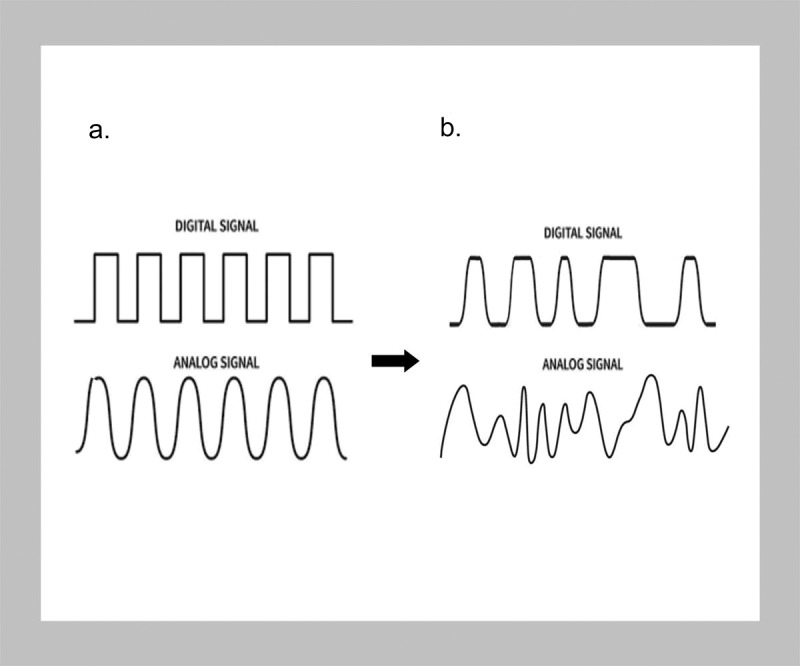
Digital versus analog information systems.

These conditional aspects of abiotic information can be conveniently represented by using the common diagrammatic representation of the differences between digital information attributable to abiotic states and analog information applied to biology. The separate information profiles between the digital and analog systems offer visual differences in these two types of information. Digital information is designed to be unerringly reproducible and consequently has a square waveform. The slopes of the analog sine wave indicate informational uncertainties and indistinctions (Figure 2). A variation to that graphical representation can now be proposed. When information in the abiotic state is partitioned between separate non-inertial quantum reference systems, the settlement of quantum asymmetries and coherences is slightly offset and no longer conforms to a strictly binary waveform. Additionally, in all living systems, analog information does not have any regular repetitions but assumes a chaotic profile mirroring the embedded uncertainties in biological information [91].

a) The digital waveform is typically depicted as a stepped or discrete square wave meant to denote its binary “bit” character, considered representative of the instantaneous settlement of electronic states or laboratory assessment of the settlement of quantum entanglements. An analog waveform is alternatively shown as a smooth continuous sinusoidal wave as harmonic averaging.

b) Entanglements in separate quantum reference frames lose their exclusively binary character, depicted by non-orthogonal waveforms. This slight skewing represents informational offsets as uncertainties, asymmetries, and inter-observer differences. In living systems, “analog” waves are characterized by ambiguities with varying amplitudes and slope profiles representing entailments as memory and self-referential inference.

Since matter and energy do not have uniform densities throughout the universe, neither does information content. Each relative consolidation of matter and energy, such as a galaxy, has its own magnetic and gravitational fields and galactic information summation. Each of these is a separate non-inertial frame of reference with respect to any other. Furthermore, each atom within that galaxy is precessing and has its own magnetic moment just as it has its own gravitational influence. Consequently, both classical and quantum interactions contribute to any universal information matrix, and both contribute to universal order. In the case of quantum entanglement, the barrier of the no-communications theorem is overcome. While it remains true that no observer can communicate individually measured information to another faster than light, in a holographic matrix, the information is already at its destination, albeit at lower density. The information to resolve the entanglement already resides in holographic N-space so that it can be instantly settled, as an entanglement communication within the universal information matrix since separateness is an illusion [48].

Consequently, relational information is the universal fabric as a constant summation of a continuous stream of reactions between energy and matter, closely reflecting theoretical physicist John Wheeler’s concept that “Matter tells space how to warp, and warped space tells matter how to move” [165]. Meaning is achieved through that relationship. Accordingly, abiotic information has its own qualities through quantum asymmetries, coherences, decoherences, and differences whose settlement has conditional attributes. Settling these conditional entanglements is an abiotic form of information measurement. In universal information space-time, there are unlimited potential information observers to settle each asymmetry in separate Markov nodal informational reference frames. No two candidates for resolving a quantum interaction need to have an identical quantum interactive status, which can be conceived as a form of abiotic measurement as a meaningful “difference that makes a difference”.

## 6. Information in the living state

Many linkages between fundamental thermodynamic processes and biological ones have been previously described [92]. Conversely, there should be biological processes that offer insight into abiotic systems. Exaptation is a well-accepted biological principle helping to account for evolutionary innovation and multicellular novelty. Some innovations arise as non-adaptive exaptations or preadaptations as a redirection of prior competencies [93]. A substantial amount of multicellular novelty represents exaptations of some prior function established at the unicellular level and redeployed to meet new circumstances [92,94–96]. For example, lens crystallins (light-refracting proteins) originated as enzymes [93]. Hence, following a principle of parsimony, the transition to cellular cognition and all life that follows must have precursor origins within abiotic states, either as an actual preexisting feature or as immanently related facets. In that case, a shared framework for universal information applicable to both abiotic and biotic systems would be highly explanatory.

In biotic states, each cell has its own local nodal Markov blanket as an individual Pervasive Information Field (PIF), a self-contained informational matrix that underlies its self-referential awareness [1,91,97]. Imputing the concept of a Markov blanket as pertinent to biology is not novel [97,98]. Kirchhoff et al. [74], p. 2) asserts that “The cell is an intuitive example of a living system with a Markov blanket.” If that Markov blanket deteriorates, it will no longer exist, and implicitly, neither would the cell. Consequently, biological systems are conditioned within a system of Markov blankets [74]. The same concept of a boundary system understood as a Markov blanket has even been further applied to Earth’s system [99]. The planetary Markov blanket separates the metabolic processes of the biosphere from the external solar environment to actively sustain a non-equilibrium steady-state through the minimization of free energy.

Markov blankets have been productively applied to understanding human cognition but are applicable across scales [100]. Notably, the compartmentalizations granted by Markov blankets in which internal states are conditionally secluded from the external environment have been used to model sentience [101]. Moreover, Markov blankets are an intrinsic part of the Free Energy Principle that has been applied to various aspects of living organisms and theories of mind [76].

Meijer and Geesink [102] have argued that accounting for the complexities of protein folding and solving Levinthal’s Paradox requires a fresh perspective on cellular information processing. How each protein is synthesized as a linear form but attains a specific, nearly instantaneous complex folded configuration within a field of billions of potential architectures and assumes a precise target configuration permitting its unique function remains enigmatic. One possibility is that each cell might store an integrated 3-D information hologram that provides a receptive memory structure that guides protein folding within that cell [102]. Importantly, each piece of a holographic image offers a particular perspective of the image but also includes the entire object. In this construct, each cell is not a stand-alone information processing unit but part of an integrated feedback information network that follows holographic principles [102]. By inference, then, a cellular PIF and N-space episenome must also have holographic features as must each of its successive iterations.

For every cell, its information matrix (PIF) integrates with its cellular senome and electrome, interacting with various internal and external electromagnetic fields [91,103]. Pertinently, the integration of the electrome has been productively modeled based on a neural network architecture that integrates information processing through an addressable holographic memory space proposed to underlie human brain activity [52,104].

The functional architecture of a cell depends on its plasma membrane, whose significant properties can be likened to a biological expression of Maxwell’s demons, assessing the information content that transits the membrane [73]. The plasma membrane sorts molecules to sustain the relative entropic status of the cell versus its external environment. Accordingly, the plasma membrane serves as an essential gateway to the cell and provides an initiating linkage across the entire senomic apparatus of that cell as a vital aspect of its cellular information management. In that way, there is orderly and preferential transit of macromolecules and energy potentials for cell–cell signaling that link to the cytoskelton complex enabling biological self-organization [105,106]. The same type of ”intelligent” excitable, gate-keeping membrane is also present surrounding eukaryotic organelles and bacteria [106].

Consequently, the flexible plasma membrane is the threshold of cellular order. Since Schrödinger’s (1944) seminal volume *What is Life* [107], the ordered cellular state has been traditionally placed in thermodynamic terms as a relatively negentropic zone in conditional, temporary violation of the second law of thermodynamics. However, the cell can now be more productively equated with informational order rather than just thermodynamic flux. The reception, assessment, and deployment of information are the cardinal features of life. Therefore, as important as the cellular membrane is for permitting ordered cellular thermodynamic flux versus the external environment as critical energy management, the flow of information is altogether as vital. Consequently, while it is true that cells are energy dissipative, they are more productively considered as equally information accumulating and dissipative. Notably, in the living frame, this information flux largely equates with cell–cell communication. What cells are explicitly dissipating is their self-referential measured assessment of environmental cues, and that communication constitutes the glue that enables multicellularity amid the complex, nested architecture of holobionic life.

Necessarily then, a key differentiating factor between abiotic states and our living privilege is coherent memory as part of cellular Effective Information (*EI) [91]. *EI is achieved when some constraints have been placed on the full range of possibilities that present to a cell from external environmental cues, achieved by its internal measurement. Multicellularity is an attempt to maximize *EI through collective assessment and communication to better sustain states of cellular homeorhetic preference. The reason for the collective assessment of information is that all biological information is ambiguous [3,91,97,108]. Any information that a cell might have has multiple sources of degradation, including time and distance from the source, various intervening media, and, most obviously, an external membranous boundary.

Consequently, all the information that any cell has is uncertain, which is why it must be measured. Shared cellular measurements of information constitute more robust information with enhanced validity (*EI) as a narrowing of any prior full range of possibilities to a smaller set that enables inference, necessitating memory [3,91]. The plasma membrane is crucial to both *EI and competent memory. *EI partially encodes as memory within that bilayer lipid barrier as lipids have hysteretic properties. Cellular memory further enacts through many other forms of physical memory storage across the entire cell within its various sub-compartments, including its cytoplasmic and nuclear (in eukaryotes) genetic complements. All aspects of the cell participate in the reception of information, its assessment or deployment, and various aspects of memory. Accordingly, cellular cognition is a cell-wide event.

Given these informational requirements of all cells, their coordinating linkage from the cellular exterior across its interior has been modeled as the senome concept [150]. The senome represents the summary architecture of all the sensory inputs that cells derive from environmental contacts through all of its sensory tools and their cross-cellular linkages. Accordingly, the senome is the means by which cognitive cells assess their environment to achieve and sustain homeorhetic balance, thus serving as a cognitive gateway. Notably, both a plasma membrane and effective memory are absolute requirements in support of senomic functions. Indeed, through this concept, it is possible to impute the existence of “senes”, which represent units of cellular experience similar to how genes serve as essential functioning sub-elements within a genome.

For example, Gatenby [169] proposes that environmental information is detected by specialized membrane-based protein gates within specific transmembrane channels through which ions such as K^+^, Na^+^, Cl^−^, Ca^2+^, and Mg^2+^ flow along electrochemical gradients, permitting nearly instantaneous cellular messaging. Two aspects of this flux are relevant. Shifts in flux status constitute meaningful information. Those shifts are dependent on a stream of information received by the cell impacting its outer membrane. Each of those environmental impacts must be evaluated and measured. Consequently, as quasi-independent events that link to an explicit further chain, those events can be considered as units of senomic experience as “senes”. However, for that to be the case and still yield productive, concordant results, a cell-wide information management apparatus is required to govern those flows of meaningful information for productive cellular deployment. The entire ensemble depends on information measured in terms of quantity and quality. Notably, the quality of information is crucial as that aspect of information is intrinsic to its validity, which links to inference and prediction upon which cellular survival depends.

Therefore, the senome represents the epicenter of the intelligent cell’s capacity to conduct the measuring assessment of its flow of ambiguous information. It directly follows that cellular-senomic memory serves as a critical component of cellular information architecture and management. All aspects of the cell contribute. For example, mitochondria demonstrate individualized reactions to environmental cues and stresses, controlling molecular cascades that link to cellular sensing [109,110]. They and other organelles integrate as coordinated senomic subsystems within the crowded, active internal environment of the cell and its cytoskeletal structures, all linking to the Pervasive Information Field (PIF) of the cell as its attachment to N-space as its Markov blanket with the plasma membrane likened to a cellular Markov boundary system. These self-directed PIFs overlap into a seamless integrated information field to coordinate multicellularity as an N-space Epipsenome, functionally acting as another Markov blanket architecture.

The N-space episenome has been previously described as a congruent information field for sharing of multicellular measurements of environmental stimuli. This encompassing multicellular informational matrix is itself proposed as a holographic projection of a field state of informational possibilities and solutions, as a partitioning of outer N-space, forming part of the memory component of the information management system of the cell [1]. Significantly, the N-space episenome serves as a heritable template for morphogenesis and developmental patterning [1,73]. As Fields and Levin ([111] p. 4) indicate, “ … … development and regeneration are the result of a collective intelligence of cells navigating the anatomical morphospace … ….” That anatomic morphospace is described as dependent on an interaction with a transcription space, controlling gene expression patterns that link to a physiological space that must also be navigated to yield physiological adaptation. In this instance, a “space” is described as “just a collection of states, together with some notion of similarity or “distance” between states” ([111] p. 4). Clearly, any such complex inter-linkages among these required spaces depend on the concordant measurement of information and its seamless coordination among trillions of participant cells. Furthermore, cells of vastly different types and even species must concurrently navigate together to maintain a functioning holobiont. Further yet, none of these cells are automatons. Each is an individual cognitive agent with its own self-identity [112]. Consequently, the need for an N-space episenome as a common informational reference platform with necessary read–write properties becomes self-evident [1,73].

Our experience with MRI *k*-space teaches that atomic precession in the rotating frame referenced to electrical gradients offers vectoral information that can become frequency-encoded spatial information to appropriate observers. In a biological context, with obligatory bioelectric fields, the N – space episenome works similarly, providing encoding spatial information to all constituent cells and underscoring its role as a morphogenetic template and developmental patterning matrix. In turn, N-space episenomes have their own outward N-space attachment as they receive coincident physical forces, such as gravitational influences or electromagnetic fields. Each separate PIF and every N-space episenome (as an aggregate of PIFs) functions as a type of Markov blanket and boundary, intersecting with others through nodal attachments but retaining features of conditional independence. From this, cells glean their spatiotemporal place, referencing their N-space episenome and its further attachment to outer N-space through the connective tissue of entanglements and precession-derived spatial cues, analogous to MRI scanning and *k*-space. Hence, the cell accumulates knowledge of the outside environment through its senome attached to effective memory, including its entire genetic complement. It further relates that knowledge to a multicellular N-space architecture as its N-space episenome.

One advantage of a universal N-space relational information matrix is that all physical forces contribute at nested levels. Accordingly, gravity contributes to abiotic systems and acts at all biological scales through local and nonlocal effects. Experiments in microgravity conditions, both in the laboratory and on the International Space Station, have confirmed that gravity is one essential component of embryogenesis. Mice exposed to space conditions in early pregnancy fail to produce viable offspring [113]. Also, there is impairment in the preimplantation development of fertilized mammalian eggs [114]. Furthermore, simulated microgravity has substantial effects on the differentiation of embryonic stem cells, with significant alterations in gene expression and signal transduction pathways [115]. Other experiments have determined that some developmental cues are derived from Earth’s gravity vector, such as the orientation of lateral branch growth in mosses [116].

Atomic nuclei have intrinsic spin and will precess along an axis if influenced by any RF field flux, resulting in an emission of RF energy to its neighbors [117]. Necessarily, this is an informational exchange and, when placed in such a context, should be considered pertinent to spatiotemporal patterning of multicellularity within a relevant N-space episenome informational matrix. Consequently, the patterning effect of bioelectric fields on morphogenesis clarifies their role in orchestrating development [118,119]. They are the essential electromic grid that consistently emerges to exert their crucial role in morphogenesis and developmental patterning from within an overarching N-space episenome. The electrome has been previously posited as heavily residing within the cellular plasma membrane as a self-generated emergent field [103]. Nonetheless, it represents a shareable information field. Since the electrome is part of the information management of the cell, any sharing sufficient to permit coordination among many types of myriad cells and other cellular species that comprise holobionic tissue ecologies obviously necessitates a common information platform, which is asserted to be its N-space episenome.

Some unusual properties of DNA are being uncovered that should be considered potential contributors to spatiotemporal patterning. It has been observed that DNA acts both as a receiver and transmitter of radio waves and other electromagnetic signals [120,121]. Castellanos et al. [122] have reported that eukaryotic transcription factors bind to target short 6–10 bp DNA recognition sites across the enormous expanse within its genome, with DNA acting as an antenna for attractional gene tracking [122]. Others have proposed that DNA acts as a fractal antenna in electromagnetic fields over a range of non-ionizing frequencies [120]. The intrinsic DNA properties of self-symmetry and electronic conduction are structural characteristics of this antenna apparatus, capable of interacting with electromagnetic fields over a wide range of frequencies [120,123]. Thus, it may not only be chemical-binding requirements that govern the helical structure of DNA.

Notably, this DNA antenna architecture is sufficiently robust that it can be technologically exploited to design an effective antenna array across a wide range of RF frequencies, particularly for applications in the terahertz and sub-terahertz ranges [124]. Correlatively, these frequencies have been demonstrated to be operative in human dermal and subdermal cells [125].

Others have observed that this same DNA architecture relates to electronic spin and its associated magnetic properties currently being investigated for use in electronic devices. Spin-based electronic charges can be exploited for the storage, processing, and transfer of information [126]. DNA is a prime candidate for mobilizing this property in living organisms because its chiral properties offer the potential to induce and control both spin polarization and spin-orbital coupling effects originating within a helical electrical field. These effects can be leveraged to permit chiral-induced spin selectivity (CISS), which has been observed in DNA molecules [127].

Any particles within a major body, such as atoms within galaxies, constituent atoms within cells, cells within tissues, or cells within holobionts, will be within a frame of reference subject to incurring electromagnetic fields. Consequently, any atomic magnetic moments will have precessional phase and frequency decay profiles based on magnetic fluxes. Therefore, along with quantum entanglements, coherences, resonances, and nonlocality, fundamental physical forces such as atomic precession are potential sources of interrelational information. However, the exact nature of those precessional interactions is dependent on the frame of reference. Even in quantum systems, it is possible to have a system with a well-defined position within one frame of reference, and well-defined motion in another, and not be in contradiction to quantum uncertainty principles since entanglements cross frames of reference [128]. Thus, all of these phenomena, both classical and quantum, can be components of information space at any and all levels, summating at reiterative levels, as abiotic Markov blankets, or as biotic PIFs and N-space episenomes, all in differential reference frames with respect to universal N-space.

In this way, based on the preceding details, the information architecture and its further information management from all potential sources guide coordinated cellular activities. It is known that cellular development is a function of migrating cells, such as the critical migration of neural crest cells. These cells are a transient embryonic cell population that migrates collectively along complex pathways to diverse embryonic locations and help establish lines of future differentiated cells in various organisms [129]. Yet, their DNA content remains constant throughout. Necessarily, the migrating cell must be attendant to various fields, such as those bioelectric fields operative in morphogenesis. However, the field itself cannot be the a priori governor of morphogenesis since those fields emerge within the process, nor are they encoded in the genome. Hence, a sensitive, preexisting multicellular spatial map is required and must exist in response to external environmental effects, including gravity.

## 7. Implications for consciousness

In view of the preceding, the nature of the self-referential consciousness embodied in the cellular form becomes a central question. The issue of how to correctly define cognition is surely contentious. No absolute definition is acknowledged. However, it is commonly considered to be a quality or state of being aware of oneself. Consequently, given the ambiguous nature of biological information as described, it is now advanced that consciousness can be considered a state of self-referential awareness in which a living observer knows that its information is uncertain. It is asserted that it is this uncertainty relationship that constitutes conscious self-reference. Consequently, the proper definition of consciousness can now be appraised as the conditional state of being aware of informational ambiguities from which self-referential inferences might arise.

Although how these qualia arose remains enigmatic and consequently, no definition will be entirely acceptable, it can still be productively proposed that this privileged status arose from within the obligate boundary/memory conditions unique to cells. Previously, this transition has been posited as a specialized thermodynamic phase transition [130,131]. Alternatively, though, and thereby consonant with the proposed definition, self-reference can now be framed as a shift in the fundamental universal information profile, whose changed waveforms and their differing slopes represent living uncertainties as informational ambiguities whose resolution requires entailing memory. As noted in Figure 2, the information profile of living information is demonstrably different between abiotic and biotic entities. Accordingly, although the origin of consciousness is not known, it can be reasonably related to the presumption of a phase transition in information quality whose instantiation requires the lipid-bilayer structure of a critical outer membrane linked to retrievable and deployable memory, thus directly relating to effective information (*E.I.) [97,112]. This conditional attribute pertinently applies to all the cellular domains that comprise all living forms. Hence, the difference between biotic and abiotic states clarifies. The living state actively manages meaningful information within the context of informational ambiguity. By contrast, abiotic particles receive information with contextual meaning and react to entanglement asymmetries within limited conditional parameters as an at-the-moment measurement by a pertinent observer/detector sufficient to represent a difference. In this frame, consciousness productively reduces to an issue of active information management in which the uncertainty relationships are resolved through retrievable memory. And of particular importance, the reverse applies. Since all living entities at all scales manage information in this specific manner, they are all conscious.

Informatively in this constitutive construct of living doubt, conscious cells both “know that they know” and “know that others know” [3]. Conspicuously, this is the background state of collaboration as the attribute of consciousness that impels shared information [3]. In this way, self-similar, self-referential organisms cooperate since they share a binding informational motif, representing a common informational substrate that multicellular organisms experience within their respective N-space boundary partitions. The abiotic state is not conscious since it does not actively manage information, merely conditionally reacts. Abiotic states lack retrievable memory, and consequently, their observer platform is constrained. Abiotic entities do not “know” that they know nor do abiotic participants “know” that others know.

There is a further notable entailment from the preceding. Since all the information that any cell has is the result of internal measurement to assess its validity, all of its information is self-produced. All cellular information is generated by its own measurement as a product of its internal and individual self-referencing [3,42]. There is a particularly important ramification to this requisite living condition. Since all information is imprecise, cells purposefully devote scant resources to its active measurement. Perfect information would require no measurement. Perfect information would confer exact cellular equipoise at all times. Consequently, all living things know “doubt” [3,91,112]. Thus, the specific context of homeorhetic balance that effectively defines the cellular state comports with doubt as a stream of uncertainty relationships. Cellular consciousness achieves biological expression as cellular homeorhetic equipoise as its state of balanced preferential flux. All cells settle informational cues to achieve their preferential state of flux balance, conditioned within the ambivalent qualities of biotic information. Notably, too, any cellular preferential state is, by definition, its most common state unless under continued stress. Consequently, since all cells are conscious, consciousness entails preference as a foundational level of cognition.

On the contrary, no non-living thing shares this circumstance. And further, doubts and preferences are valenced experiences at scale since they directly pertain to one particular state versus another. Consequently, all living things at all scales have experiences and subjective feelings of their own circumscribed type. By contrast, non-living things have a much more limited observer capacity, detecting conditional entanglement asymmetries as differences that are settled at the moment with no entailing effective memory that pertains to a next state.

Pertinent to abiotic states, when information is placed in the rotating frame, independent observers will receive “off-set” information compared with one another, which has also been confirmed within quantum reference frames [90]. This discrepancy creates inherent informational asymmetries. In separate rotating frames, the relational information between any two particles with respect to any other particles or sets of particles will be slightly variant as an asymmetry. One of the reasons for this phenomenon, though typically applied to the study of large masses, is that any rotating frame exerts a frame-dragging effect on others that affect measurements, known as the Lense-Thirring Effect, an embedded feature of space-time predicted by Einstein’s General Theory of Relativity [132]. Consequently, these can become differences in entanglements between otherwise identical observers in separated rotating frames with those differences representing information [133].

Measurement degradations are known to be problematic in quantum reference frames, described as measurement back action, limiting the frame’s usefulness and requiring error corrections [134]. Furthermore, within rotating frames attaching to a universal referencing system (holographic N-space), any particles in separate rotating frames experience slightly different entanglement asymmetries and relational correlations. This interrelationship can now be considered a form of conditional independence. In this framework, the settlement of conditional entanglement asymmetries become “differences which make a difference”. The resolution of these quantum uncertainties maintains the structural order of the observer/detectors’ relevant Markov blanket and boundary and simultaneously contributes to the universal holographic N-space.

This background is pertinent since it is being specifically argued that these differences represent “meaningful” abiotic information, and further, this condition arises due to N-space partitioning, thereby representing the exaptive ground state that eventually licensed cellular self-referential consciousness. Accordingly, the living state enacts through effective biotic circumscription of N-space as a system of nested Markov blankets and boundaries. The crucial difference between abiotic and biotic states is the resonating biological boundary and its embedded retrievable memory, enabling what might be considered a tightly bound and coordinated Markov blanket as a non-equilibrium steady state, permitting active information management. This system offered the necessary constraints on information space-time that self-reference with its inferential implications requires. Correspondingly and of specific importance, living boundary conditions enforce that all information is self-produced and self-consonant, thereby defining the self-referential state. Thus, the circumscription of N-space that constitutes the information architecture of a cellular PIF is a Markov blanket-membranous boundary system linked to effective retrievable memory, permitting the minimization of free energy for the limitation of perceptual error and suppression of surprisal according to the free energy principle (FEP). It follows that the cell is the physical embodiment of these attributes and is thereby the fundamental effective unit of consciousness.

The concept of separate quantum reference frames is becoming a specific feature of modeling living systems. Fields et al. [135] consider neural actions as hierarchies of quantum reference frames. Within that cohering model, sub-networks of neurons can “compute local logical operations” in which neurons are not just biological wires since that simplistic conception offers no explanatory information about how reality is encoded. Instead, their actions are a total functional complexity within multicellular microenvironments developing within a context of a “neural primordia”. Under the constraints of FEP, finite living systems with limited free energy evolve toward a “neuromorphic morphology” as a developmental architecture linking neuronal intracellular signal transduction to the cell–cell signaling necessary to permit the multicellular activities and perceptions of the mammalian brain [136].

Accordingly, neurons and their linked biological structures interpret physical stimuli through measurement, mirroring the active-inference principle and comporting with quantum information theory and quantum reference frames as central to a biological means of calibrated (referenced) measurement [135]. A notable feature of quantum reference frames deserves emphasis. Each is its own reference system for measurement. Any attempt to transfer a quantum reference frame to another separate one can only be successful if the other quantum reference frame is functionally equivalent [135]. Consequently, quantum reference frames can only usefully exchange information if there is a shared platform for referencing measurement; hence, the requirement for an overarching N-space compartment that permits that mutual assessment, enabling collaborative life among trillions of cells as a series of nested tissue ecologies, and perhaps also significantly relates to universal structural order.

Similarly, the concept that life is autopoietic is not novel. Maturana and Varela considered cognition as an autopoietic system that could self-regulate through distinct self-regenerating interactions with the environment [137]. Cárdenas-García [42] has elaborated a crucial concept of the living self-production of information, termed “info-autopoiesis”. Every cell creates its own reality; consequently, each has internalized experiences that can never be completely identical to any other. This posture is necessarily the case since the actuating cusp of self-reference is an individual cell’s measurement of information, settling perceived ambiguities into individually derived exclusive patterns of bioactive deployment. Thus, any stimulus crossing a living membrane is an experience since it entails a defining interaction that is entirely self-contained before its external communication.

Moreover, whatever interprets that communication is obliged to do so through its individually self-generated production of its information content and measuring value. However, and of notable discriminatory importance between the animate and the inanimate, it is only in the living state that a predicted stimulus that did not occur could have pragmatic meaning. It is only in the living, conscious state that meaningful information can be derived from “a difference that is’ *not* a difference”, i.e. self-referential awareness of a self-generated prediction of an expected event that has not occurred. As keenly articulated in a 2022 personal communication from physicist Chris Fields, “The information encoded by an electric field “makes a difference” to an electron if it changes its momentum. What the electron lacks is memory for its previous states.”

Derivative within the above, and as noted previously, any informational stimulus that crosses the cells’ outer membrane links to an entire senomic apparatus for requisite internal measured assessment of its validity to sustain cellular self-generated states of internally measured experiential preference [2,73]. As a compulsory pathway, it becomes self-evident that there is no “hard problem” of consciousness. All information has an obligatory set of senomic linkages, and everything that impacts the cell membrane and becomes internalized is an experience with both quantitative and qualitative subjective aspects. The self-production of information ineluctably includes all fundamental aspects of sentience as conscious self-reference since all are necessary for cellular problem-solving [2,3,73,138,139].

This living requirement for the self-production of information helps explain many physical phenomena with no known genetic correlate. For example, a few individuals lack a perceived capacity for visual imagery (aphantasia), remaining unable to “see” things with their minds [140]. In contradistinction, others experience extremely vivid imaging sensations (hyperphantasia) with daydreams and nocturnal dreams as intense as seeing. Although some studies with fMRI suggest differences in connectivity between the prefrontal cortices and the visual system, the actual cause is undetermined. Clearly, each of us experiences the environment in an entirely personal manner, and language can only approximate these exact experiences but never equal them.

There is a specific and consequential derivative about how to conceptualize N-space partitions when it is realized that the ambiguous nature of information and the boundary system of the cell defines each cell’s reality through the self-production of its knowable information. In universal relational N-space, the universe is information, and information is reality. It follows that any partitioning of N-space into Markov blankets and boundaries creates its separate subset of reality.

In the living frame, each partition of N-space is reality within its nested architecture. For a cell, its PIF as its Markov blanket is its field of possibilities of informational cues from which it can derive its internally generated, measured interpretation. Overlapping PIFs summate into a conjoining N-space episenome information field that permits concordant measurement of the sum total of multicellular environmental cues as multicellular reality. Consequently, our reality is the partition of N-space that we are equipped to observe and measure. Since in the living frame, the N-space episenome is information space encoded in heritable and retrievable memory, multicellular reality is self-similar through genetic reproduction since genes are memory linked to information space. Hence, every organism is a series of nested Markov blankets and boundaries as a consonant node of partitioned universal N-space, representing an effective circumscription of N-space available for observation by that organism. Hence, different senomic apparatuses that link to their respective PIFs and N-space episenomes offer different realities from within universal N-space. Your partition controls your reality. Accordingly, species are concordant N-space partitions for the measuring assessment, communication, and deployment of information for problem-solving through their similar senomic architecture.

Accordingly, the best way to consider information is by focusing on the specific attributes of the information observer in any particular context. When that stance is adopted, the essential differences between abiotic and biotic states clarify. The living state of homeorhetic flux requires cellular boundary conditions and effective memory, creating a more ordered information state inside the cell than the outer environment. These conditions maintain the cellular self-referential information management system, forming the prerequisites of consciousness. Abiotic states can detect relevant information to settle entanglements and sustain quantum correlations but cannot order information beyond contributing to a quantum reference system and universal N-space.

The same framework offers clarity about the contentious issue of plant intelligence. It has been asserted that plants are electromic interfaces from which intelligent behaviors emerge [141]. In complex multicellular organisms like plants, electromic activity could only be productive if placed within a global state of receptivity that references an information standard from which co-aligned measurements can actuate, thereby enabling learning, effective memory, and intelligent organism-wide behaviors and responses to stress. In sum, plants must be “knowledge-accumulating systems” [141]. Absent an N-space episenome as a conjoining information platform that permits the narrowing of an unlimited palette of implicates into a circumscribed range, concordant multicellular life could not occur. Accordingly, plants are compartmentalizations of N-space with a crucial plasma membrane that links to retrievable, deployable memory. Furthermore, those cells are self-referencing, permitting effective information management for cellular problem-solving. Hence, plants are conscious, intelligent agents.

The N-space episenome represents a compacted yet entirely cohesive informational landscape permitting shared measurement to sustain multicellularity and serving as a component of multicellular memory. In that way, it becomes a developmental canvas with embedded constraints as well as a propulsive agency as a field of possibilities. Thus, it represents a summation of developmental Bohmian implicates as appraised by intelligent cells. Thus, level upon level, an entwining of self-produced information and communication becomes the dynamic that enables life through its reiteration at all scales [12]. Consequently, the phenomenon of self-referential cognition, melding biological systems within a universal informational construct, reaches toward the philosophical notion of “being-in-the-world” [12].

Markov blankets are considered “ubiquitous features of living systems” as necessary for any living entity, such as a cell, to distinguish itself from its environment [142]. Therefore, the cell and even its internal organelles are considered various iterations of biological Markov blankets, representing a scale-free architectural element that reiterates to the level of the Earth’s biosphere [142]. At each level, a relational Markov blanket establishes sufficient conditional independence from the environment to maintain self-contained homeorhetic equipoise.

Notably, any dynamic system that is equipped with a Markov blanket, representing a statistical separation between an internal state and its environment, attains a status that can be interpreted as typifying a nodal type of Bayesian inference about that external environment, thereby implying probabilistic information about what is external to its boundary [143]. The applicability of Markov blankets to abiotic structures is based on the previously described entailing characteristics of abiotic information, such as hysteretic properties, Lense-Thirring effects, and precessional relationships with time-decay curves acting as nominal projected information trajectories. Although the concept of Markov Blankets has been restricted to biological systems, when this theoretical construct is placed in a universal N-space framework, it can helpfully account for the attributes of conditional independence of major physical structures across the universe as a contributing factor in universal order.

Placing abiotic states within the model of a Markov blanket and boundary, which has weakly coupled and random dynamic features linked to uncertainties and some degree of conditional independence, permits the application of the Free Energy Principle (FEP) to abiotic states as a type of self-evidencing [144]. In FEP, the minimization of variational free energy corresponds to a decrease in surprisal as inferential self-information, whose upper boundary represents the Bayesian prediction error. Fields et al. [142] placed FEP into a space-time setting as a scale-free quantum information theory in which quantum systems can be regarded as observers sufficient to imply meaning to observational outcomes. Further yet under the free energy principle, random dynamical systems assert a kind of self-evidencing whereby even generic quantum systems can be regarded as observers with assignments of meaning to observational outcomes [145]. If this is accepted, a reconciliation between abiotic and biotic observer states opens.

Many philosophers and scientists have insisted over centuries that the living circumstance must relate to a preexisting and encompassing universal sense-awareness, ensuring that everything has intrinsic connectedness [108]. For example, Alfred North Whitehead [146,147] argued that quantum space-time was the enfolding of implicates connecting every object to every other universal participant, including the inanimate, through an embedded experiential component as universal sense-awareness. However, the novel frame that is being offered imposes a caveat. That concept of universal sense-awareness need not have any necessary implication of universal consciousness. Instead, universal sense-awareness in abiotic states relates to the limited receptor palette of abiotic informational participants, which are more than previously considered but still substantially circumscribed compared to living consciousness.

Self-reference requires living information storage and retrieval as a second-order form of memory beyond hysteresis in the physical realm. The abiotic observer interprets its information field as interrelational asymmetries and coherences but is divorced from effective retrievable memory. The gap can be conceptualized as the difference between an experience as instantaneous meaning in the moment (such as relational position or momentum) and an experience with the qualia of overlapping memory of prior experiences. Indeed, in the abiotic realm, quantum superpositions of implicates settle into physical/energetic explicates. They do so instantaneously or nearly so since they exist within the moment. Living systems do other things, settling their implicates through entailing memory with its embedded time lag in which its past looms as stigmergic interactions between the observer and its environment [142].

When cellular functions are placed in this context, it becomes obvious that genes are tools of cells as a vital aspect of their total effective, organized memory as a record of previously settled implicates [91,112]. Genes are records of successful cellular problem-solving. However, cellular problem-solving is cognition in action. Hence, from the foregoing, the origin of cellular self-organization clarifies. Self-referential cognition is self-organization [1,3].

Biology is recognized as a series of exaptations or leveraged pre-adaptations as a consistent form of reiteration and mosaic formulations. Furthermore, by the principle of optimality, any further steps in such reiterating processes must remain adherent to its initiating premises to follow an optimal path [148]. For biological entities to successfully build productive outcomes within a state of conditional ambiguity, there should be a precursor that could represent a substrate for that necessary exaptation. Thus, the measurement of abiotic informational asymmetries that contribute to universe order forms the necessary precursor for the living measurement of biological informational ambiguities.

**Figure 3.**
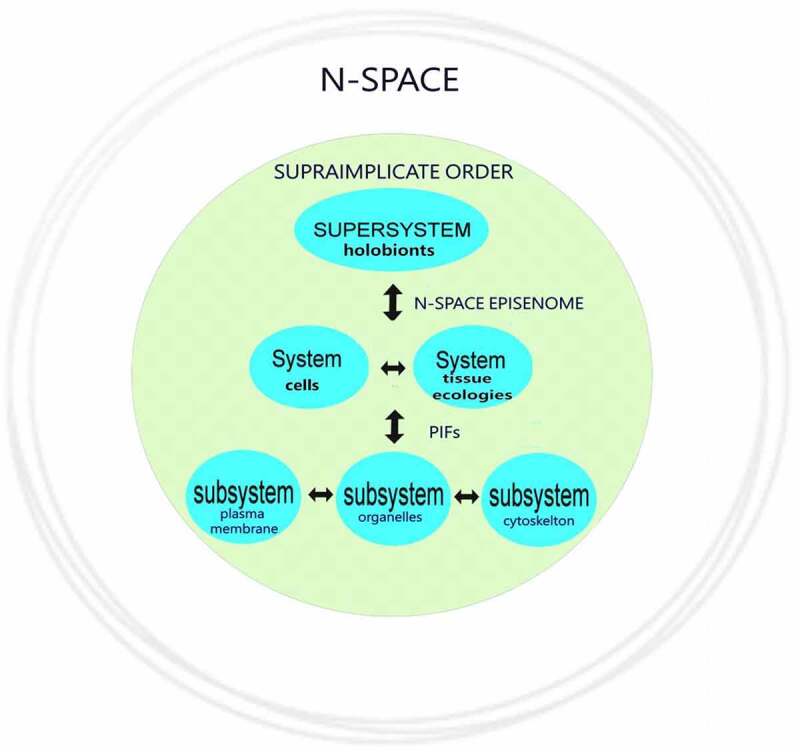
The reiterating system of systems/Markov blankets within living systems.

Fields et al. [142] indicate that “any interaction between two finite quantum systems in a separable joint state” can be interpreted as classical communication. That framework further asserts cellular interactions with the environment can be considered meaningful information since it derives from cellular measurement with respect to some reference frame [142]. Fields et al. [142] also notes that this circumstance is actually no different than the process for determining whether the spin of a qubit is up or down. Therefore, a system of compartmentalizations of a universal N-space matrix, each segregated from others according to the model of conditional Markov blankets and boundaries, permits meaningful informational associations as correlative relationships. That meaning is derived from the obligatory interrelationship between particles and energy and their embedded informational trajectories within an overarching informational lattice. Each such interaction contributes to holographic N-space through entanglements and precessional resonances. In this manner, informational nodes as localized collections of matter and energy can represent an informational architectural matrix, whether in abiotic states, cellular PIFs, or N-space episenomes. In both systems, all compartmentalized nodes, no matter how many intermediaries, attach to outward N-space, forming a Bohmian subsystem–system–supersystem as a systematic nesting of corresponding Markov blankets that reiterate across both abiotic and biotic states that links to conscious states (Figure 3). Thus, the living state is the one in which information achieves causal efficiency over matter [131]. Consequently, an equivalency is reached. Consciousness and life are an identity [73,97] [138,139,149].

Subsystems of intracellular structures and the plasma membrane permit intracellular informational management as individualized pervasive information fields (PIFs) and localized tissue ecologies as coalescent Markov blankets at the systems levels. These combine at the supersystem level as the relevant cellular N-space episenome, as reiterating supersystem Markov blankets that represent the informational platform for coherent shared cellular information of environmental information and serving as a heritable template for morphogenesis and developmental patterning. Supersystem N-space episenomes (holobionts) experience a supraimplicate order that engages surpacellular consciousness, permitting collective cognition in confrontation with environmental stresses.

Derivatively then, the basal aliquot of consciousness that is instantiated within every cell systematically reiterates level by level to ultimately form our idiosyncratic manifestation of human consciousness, which can further project as a supraimplicate realm of collective consciousness as a next reiteration [2].

Accordingly, the crucial difference between abiotic and biotic states crystallizes. That differential crux is the specific attribute of the observer/detectors of universal information that defines the characteristics of their applicable N-space compartmentalizations. By virtue of the discrete, intelligent boundary of the senomic cell membrane acting as a quasi-Maxwell’s demon enacting internal informational order, the living state stands categorically apart from abiotic Markov blankets lacking interactive boundaries. Hence, the cell membrane is fundamental to cognitive life [2,150–152], enabling self-reference and accounting for the imposition of the self-production of information within cellular boundaries. The senomic cell membrane linking the cellular senomic apparatus to all the cellular forms of effective, retrievable memory stimulates conscious self-reference and creates living experientiality as a cellular embodiment capable of the minimization of quantum prediction error. It is at the cellular level, and only there, that there is the sufficient co-alignment of the relevant informational reference frames granting the necessary resonances, reciprocations, and coherences as conscious self-referential quantum computation that energizes its function as a communication resource [142].

The preceding discussion has a significant ramification. As boundary conditions explicitly relate to consciousness, there is no universal consciousness as we have typically conceived that notion. The outer universe contains an infinitude of connections through local and nonlocal entanglements, which have both local and nonlocal consequences. These can arguably be equated with the universal sense-awareness many scholars have defended. However, in and of themselves, these connections are not consciousness or even proto-consciousness. Instead, they are quantum correlations as their own form of universal connectedness. Consciousness only occurs within reverberating boundary mechanisms embodied in cells that permit self-referential quantum coherences. The abiotic realm has much more circumscribed informational meaning. The informational difference between the two relates to the nature of the observers and their capacity to structure that flow of connected information, not the source of information per se. As Jon Lieff [153] aptly notes about living systems, “Although cells are considered the most basic characteristic that defines life, it is actually the conversations among cells, and also the conversations that take place inside them, that determines biological activity and produces the essence of life.” That illuminating sentence would be just as correct if the word ”consciousness” was substituted for “life” since life equals consciousness.

De Loof [103,154,155] had presciently emphasized that life is a verb, not a noun.

When it is fully recognized that only the correlations between matter and energy can be measured, then the entire universe is a verb, not a noun.

When an assertion is made that consciousness is an intrinsic property of all cells as the self-referential awareness of ambiguous information, then an integrated information theory is implied. Integrated Information Theory (ITT) offers a productive framework when applied to cells and neatly interfaces with a theory of mind [156]. Simply put, a theory of mind is cell theory. Although there have been many modifications of ITT over time, five “self-evident” truths about consciousness are distilled into its current framework [157]. Those five axioms indicate (a) consciousness exists, (b) consciousness is structured, and every experience is a combination of its aspects, (c) consciousness is informative, allowing the differentiation of one experience from the particulars of other possible experiences or those of others, (d) consciousness is integrated and to such an extent that each experience is not reducible to its “interdependent components”, and (e) each experience is “exclusive” and unlike any other.

Notably, all of these meld well with the base parameters of a cell theory of consciousness and clarifies the base differences between abiotic and biotic states. Abiotic states without the boundaries necessary for planetary life do not have a functioning apparatus to appraise information at sufficient depth to allow the necessary integration, exclusion, interdependence, and structure that consciousness demands. On the other hand, those aspects of information management are present within the cell. However, information is still meaningful and informative in abiotic states since it has observer/participants that settle asymmetries through N-space interrelationships. Consequently, abiotic states have limited informative connectedness. The crux of the difference that characterizes the living state is the concept of knowing self-reference, which requires boundaries circumscribing a relevant N-space compartmentalization. In the abiotic case, information is objective and can stand apart from knowledge, at least in some applications. Thus, abiotic information also represents a “phenomenon that in some sense exists independently of one’s knowledge” yet retains some element of causality [158]. However, none of the other knowing particulars of information quantity and quality exist outside of the cell; on the contrary, all apply within cellular boundaries.

Consciousness exists within cells since cellular information is structured, valenced, separated, self-referential, integrated, interdependent, and exclusive. By definition, every cell has self-generated information; therefore, all attendant experiences must also be exclusive. Furthermore, multicellular life, with a brain or not, is completely dependent upon the seamless integration of information among trillions of participants. Every cell has its basic aliquot of consciousness that can summate in idiosyncratic combinations through their individual N-space episenomic attachments, becoming all the varieties of macroorganic consciousness that we can observe, including our own [1,2]. Pertinently, recent research indicates that individual neurons participate in “mind” with distinct firing patterns within the dorsomedial prefrontal cortex, contributing to an individual’s inferences about the thoughts of others [159]. As the authors conclude, individual cells are central to any theory of mind.

The specific advantage of applying an informational field matrix to biology is that it suitably matches living processes. In conscious operations, there is no precise localization of functions and no concomitant burdensome energy concentrations in a single location. This phenomenon has already been validated through neuroimaging. Experiments have demonstrated that the spatiotemporal distribution of neural activity from a sensory stimulus does not relate to any single brain locus, whether for sight, hearing, thinking, or dreaming [160]. Instead, representation is simultaneously distributed among different brain centers, naturally targeting some specific brain loci more than others through specialized cells such as those within the visual cortex. However, it is the information-based interaction among widely distributed brain loci, i.e. the exchange of pragmatic, effective information that represents the specific relationship that *becomes* the experience. Notably, this specific neuroimaging feature forms the basis of the Global Neuronal Workspace Hypothesis that explicitly acknowledges the diffuse nature of consciousness [161]. Consequently, any mental image or experience is not embodied within the neuronal actions per se. Instead, it resides within the dynamic distribution of neural impulses.

Guidolin et al. [170] offer an insightful perspective on conscious functions noting that effective information management requires mapping between the external environment and internal cerebral cell-based activities. However, even though cell-centered, the final map must represent a “holistic assembly of a multilevel functional organization.” In this manner, relatively compartmentalized, modular zones of brain activity are not merely arranged in parallel or hierarchically but are nested within each other. Necessarily, though, any such final map requires a reference point for concordant measurement conforming to the N-space model with its relevant read–write partitions. Nested modular zones must interconnect but remain conditionally independent mirroring Markov blankets. All information within a “holistic assembly” comports with a Bohmian multilevel enfolding of implicates.

Some will contend that quantum mechanisms are being used as a catch-all concept to fill in the blanks in our knowledge of our universal system. Undoubtedly, though, quantum mechanisms are real, and unquestionably, they also demonstrably contribute to living experiences across all scales [162]. Thus, it is productive to consider information as a superposition of possibilities whose settlement constitutes a difference, which plainly describes our living circumstance. In that case, the Bohmian superposition of possibilities inherent to quantum relationships reflects the inherent ambiguity of biological information, necessitating its measurement, thereby defining the self-referential experience.

## 8. Conclusion

Information is the interrelationship between matter and energy, forming the connective tissue of a holographic universal relational information matrix (N-space). Within this matrix, widely separated physical structures such as galaxies can be regarded as partitioned nodal information compartments within that universal N-space informational matrix. These partitions represent zones of relative compaction of information density, conceptualized as separate Markov blanket and boundary nodes. Each of these partitions is conditionally independent of others as separate non-inertial quantum reference systems that further connect to a universal relational information fabric. When entanglements and quantum correlations are placed in separate rotating quantum reference systems, entangled particles within separate systems constitute distinctive referential observers that may conditionally settle entanglement properties. These conditional off-sets between entanglement observers represent an abiotic form of measurement in context, constituting meaningful information as a “difference that makes a difference” both within the nodal partition and concomitantly in universal N-space. Consequently, every abiotic interaction, whether due to classical Newtonian or quantum forces, contributes to local order within a relevant Markov blanket as a separate informational partition and to universal order within a holographic N-space universal relational information matrix.

This set of conditional interrelationships based on N-space partitioning provides a prevenient, exaptive channel toward effective information management in biotic states, chartering living self-organization. Biological organization conforms to the same reiterating system of interconnected N-space partitions as Markov blankets and boundaries that link intracellular organelles to cells and cells to multicellularity, holobionts, and collective macroorganic activities. Consequently, living systems consist of a reiterative interconnected ensemble of Markov blankets, in which some degree of conditional inference pertains at every nested level [74,76]. Significantly, biological organization becomes a function of learning to predict how environmental shifts will affect its relevant Markov blanket as its pertinent information space. Clearly, that type of living self-organization is dependent on competent boundaries that separate internal from external states [151].

In this manner, the limitations of classical systems theory are overcome. As Heylighen ([163] p.3) emphasizes, when complex systems and self-organization within those systems are integrated as cognition, we can arrive at an entirely different biological philosophy, which views “the essential building blocks of the universe as actions and interactions, rather than as pieces of matter or energy.” Intelligent self-organization is a product of this universal interactive matrix wherein the matrix is directly understood to represent the connections between fundamental matter and energy.

Nonetheless, there are consequential differences between biotic and abiotic systems. Abiotic states do not have discrete, effective boundaries like the cellular plasma membrane nor retrievable memory. Accordingly, physical particles have entanglement connections and contribute to universal order both locally and at a distance across the universal N-space informational fabric but do not have self-referential consciousness. They can measure but only to the limit representing the conditional settlement of uncertainties, leading to an at-the-moment next state. On the other hand, self-referential consciousness and sense-awareness require senomic membranous boundaries and the liberties and constraints of the ordered flow of internal information within a discrete living N-space partition that depends on retrievable and deployable memory. Conspicuously, that internal self-referential measurement links to preferential states that reflect internal doubt, prediction, and problem-solving, standing apart from abiotic systems.

Within the cellular boundary system, all entailing aspects of consciousness are instantiated within the basal cellular form. All the reverberations between the cellular senomic membrane and the plethora of interior cellular tools are experiential. Georgiev ([164] p. 2) illuminatingly articulated this principle, “The essence of consciousness is the act of experience.” Although true enough, an amendment is required. Consciousness is the act of experience within deployable memory. Doubt, preference, and prediction all require contextual memory. Thus, the crucial transition of the quality of information from conditional entanglement connections to the self-referential state depends on the entire senomic apparatus, which connects memory to inference to effectively settle uncertainties. The living state embodied in the cellular form mandates that all the information that any cell has is self-produced through internal measurement. Accordingly, consciousness is self-referential specifically because it is internally self-generated as an internal measurement. Hence, information is universal. Consciousness is local.

Measurement is our only way of learning. The constitutive universal uncertainties mandate measurement. The process of the measured assessment of ambiguous information is how our cells have survived on this planet for at least 3.8 billion years. However, that embodied facility that successful perpetuates planetary life over billions of years in the cellular form must be a ramification of a universal enfolded structure and cannot be its exception. Consequently, a universal relational information matrix (N-space) exists to enable the concordant measurements that permit the living state. If measurement is applicable within the living state, and that living state is derivative of universal processes, its origin must be a repercussion of a precedent universal abiotic properties.

Information is everywhere as fundamental universal action potentials. Its meaning is a function of its detector’s context. Consequently, life is a verb because information is a verb. Its meaning is inextricably linked to any next state. And since the universe is information, the abiotic realm can also only be grasped as a verb, not as particles. Our reality is not the fundamental particles that physicists catalog. Instead, we exist among the foundational universal informational interrelationships in which elemental particles participate.
